# Genomic diversity of SARS-CoV-2 carriage in a cohort of schoolchildren in Côte d’ivoire during COVID-19 pandemics: insights from pre-delta emergence

**DOI:** 10.1186/s12879-025-12374-4

**Published:** 2026-01-08

**Authors:** Kouassi Firmin Missa, Kanny Diallo, Kolotioloman Jérémie Tuo, Kouakou Brice Bla, Tiémélé Laurent-Simon Amoikon, Kossia Debia Thérèse Gboko, Amelan Marie-Flore Didia, Ganna Kovalenko, Biego Guillaume Gragnon, Joyce Mwongeli Ngoi, Ian Goodfellow, Robert Wilkinson, Gordon Awandare, Bassirou Bonfoh

**Affiliations:** 1 Centre Suisse de Recherches Scientifiques en Côte d’Ivoire (CSRS), Abidjan, Côte d’Ivoire; 2 West African Centre for Cell Biology of Infectious Pathogens (WACCBIP), University of Ghana, Accra, Ghana; 3 Biology and Health Laboratory, UFR Biosciences, Félix Houphouët-Boigny University (UFHB), Abidjan, Côte d’Ivoire; 4 Laboratory of Microbiology, Biotechnology and Bioinformatics, Félix Houphouët-Boigny National Polytechnic Institute, Yamoussoukro, Côte d’Ivoire; 5 National Laboratory for Agricultural Development Support (LANADA), Regional Laboratory of Korhogo, Korhogo, Côte d’Ivoire; 6 The Francis Crick Institute, London, NW1 1AT UK; 7 Department of Infectious Diseases, Imperial College London, London, W12 0NN UK; 8 Wellcome Discovery Research Platforms in Infection, Centre for Infectious Diseases Research in Africa, Institute of Infectious Disease and Molecular Medicine and department of Medicine, University of Cape Town, Observatory, 7925 Republic of South Africa; 9 Division of Virology, Department of Pathology, University of Cambridge, Cambridge, UK; 10 Pierre Richet Institute of Bouaké, National Institute of Public Health, Bouaké, Côte d’Ivoire

**Keywords:** SARS-CoV-2, RT-qPCR, Genomic, Mutation, Oxford Nanopore Technology, Abidjan, Korhogo

## Abstract

**Background:**

After the first case of COVID-19 was reported in Côte d’Ivoire in March 2020, the virus spread significantly, with several epidemic waves. During a carriage study conducted from November 2020 to April 2021 to examine the oropharyngeal microbiome of school children, the presence of several other pathogens was investigated. This study characterised the diversity of SARS-CoV-2 detected in a cohort of school children in Côte d’Ivoire.

**Methods:**

Oropharyngeal swabs from participants in Korhogo (*n* = 37) and Abidjan (*n* = 39) were analysed. RNA was extracted from the samples, followed by RT-qPCR detection of Coronaviruses. Sequencing was done on an Oxford Nanopore platform and data analysed in GISAID.

**Results:**

Out of 445 samples collected, 15 (3.37%; 5 in Abidjan and 10 in Korhogo) tested positive for SARS-CoV-2 and were sequenced. Genomic coverage of over 70% was obtained for 12 genome sequences (80%). There was a significant difference in SARS-CoV-2 carriage over season per sampling visit (*p* < 0.05). The variants identified were of type Eta and Beta, including variant of concern (VOC) B.1.351 (6.7%), Variants of Interest (VOI) B.1.525 (60%) and other unclassified lineages B.1.1.318 (6.7%), A.19 (13,3%) and A.27 (13,3%). D614G mutation (*n* = 11, 73,33%) located on the S gene was the most common, followed by the T205I (*n* = 8, 53,33%) located on the N gene in this collection of genomes.

**Conclusions:**

This study highlights the diversity of SARS-CoV-2 that circulated in a cohort of children in Côte d’Ivoire and reports carriage of the A.19 lineage, a variant which circulated more frequently in West Africa during the study period.

**Clinical trial number:**

Not applicable.

**Supplementary Information:**

The online version contains supplementary material available at 10.1186/s12879-025-12374-4.

## Background

Since the beginning of the COVID-19 pandemic in December 2019, more than 770 million people have been infected worldwide and variants of SARS-CoV-2 have appeared on several occasions [1]. Certain variants have spread throughout the world and have made major contributions to the various waves of infection that have occurred in different regions [2].

The increase in cases of new strains and the resulting waves of infection mean that there is a need to assess the preparedness of public health institutions with limited resources, including in Africa, to limit the impact on community transmission [3]. As of December 5, 2025, the cumulative number of cases of COVID-19 in Côte d’Ivoire, a country located in the inter-tropical zone of West Africa, is around 88,600, with 835 deaths according to the WHO [4]. As an economic centre of francophone West Africa [5], the country receives numerous visitors from neighbouring countries and Europe, making it an ideal location for cross-border transmission of pathogens. The first case of COVID-19 was reported in Côte d’Ivoire on 11 March 2020, the virus then spread significantly in the country, with several epidemic waves [6].

The potential role of children in the transmission of COVID-19, particularly in the school environment has been debated worldwide. Although children generally have a lower incidence and less severe forms of COVID-19 than adults, the increasing proportion of paediatric cases and their potential contribution to transmission of the virus has highlighted the importance of considering their inclusion in COVID-19 vaccination programmes [7]. However, a limited number of studies have focused on genomic surveillance of the virus in children. In Côte d’Ivoire, most of the cases identified were in adults [8]. The spread of the SARS-CoV-2 virus is known to be mainly due to transmission by asymptomatic carrier, a phase of infection that may go undetected. The difficulty in tracking these carriers complexifies the control of variants’ spread. Global genomic surveillance is therefore essential in the fight against current but also future epidemics [9].

Most mutations in the SARS-CoV-2 genome occur during viral replication. The resulting mutant viruses are then subject to selective pressures within the host and/or during human-to-human transmission [10]. Whole genome sequencing (WGS) is therefore essential to track genomic changes in the virus, and can help improve surveillance of epidemics. One of the difficulties in controlling the transmission and severity of COVID-19 worldwide is the emergence of SARS-CoV-2 lineages and sub-lineages (variants) with increased transmissibility [11]. SARS-CoV-2 lineages have been defined as variants of concern (VOC) or variants of interest (VOI) by the WHO [12]. The variants of concern (VOC) include alpha, beta, gamma, delta, and omicron, with their respective lineages [13, 14]. Variants of interest (VOI) are eta, iota, kappa, lambda, epsilon, zeta, theta, and Mu [13].

Genomic surveillance of SARS-CoV-2 is crucial to detect viral variants threatening the management of COVID-19. Today, beyond simple PCR detection, most countries are able to sequence SARS-CoV-2, thanks to significant investment in infrastructure and capacity building [15]. This study therefore provides a characterisation of SARS-CoV-2 virus variants detected retrospectively in a cohort of school children from the north and south of Côte d’Ivoire.

## Methods

### Study sites and participants

As part of a carriage study exploring the microbiome of schoolchildren in Côte d’Ivoire, multiple pathogens were tested, including SARS-CoV-2, *Haemophilus influenzae* and *Streptococcus pneumoniae*. This study is a subset analysis of SARS-CoV-2 positive samples detected in the paper Missa et al., 2024 [16]. Briefly, a cohort of schoolchildren aged 8 to 12 was recruited and followed up from November 2020 to April 2021 in two primary schools in Korhogo (*n* = 37) and Abidjan (*n* = 39). The study was conducted of a period of 6 months with monthly samples collection surveys (S1-S6), with a total of 445 samples collected. Ethical approval was obtained from the National Ethics Committee (IRB000111917) [16].

### Sample collection and RNA extraction

Oropharyngeal samples were collected monthly from each participant using sterile swabs. Swabs were stored in 1 ml RNAprotect to protect RNA stability, and stored at -80 °C [16].

RNA was extracted from the oropharyngeal samples using the QIAmp Viral RNA extraction kit (Qiagen, Hilden, Germany) following the manufacturer’s instructions. RNA samples were quantified using the Qubit RNA HS Assay Kit on a Qubit 4 Fluorometer (Thermo Fisher Scientific, MA USA) [16].

### Coronaviruses RT-qPCR

Coronaviruses were detected in samples by RT-qPCR as described in Missa et al. 2024 [16]. Briefly, a 20 µL reaction mix was made containing 10 µL of Luna Universal Probe One-Step Reaction Mix (2X), 1 µL of Luna WarmStart RT Enzyme Mix (20X), 0.8 µL of each E Sarbeco Forward and reverse primer (10 µM), 0.4 µL of E Serbeco Probe, 5 µL of nuclease-free water and 2 µL of RNA. RT-PCR was performed using the Bio-RAD CFX96 machine according to the following amplification programme: a reverse transcription step at 55 °C for 10 min, followed by initial denaturation at 95 °C for 1 min. Amplification was then carried out over 45 cycles, including denaturation at 95 °C for 10 s and an elongation phase at 60 °C for 30 s with plate reading.

### Library preparation and sequencing

Genomic sequencing was performed using the ARTIC LoCost ncov-2019 sequencing protocol with primers (ARTIC V4.1) [17].

The final library, containing barcodes assigned to each sample, including two negative controls was prepared through the ligation of ONT sequencing adapters, quantified using Qubit, and loaded onto a new R9.4.1 flow cell.

### Genome assembly

The ARTIC bioinformatics protocol was used to generate consensus sequences by aligning the sequenced amplicons from each sample to the reference genome (Genbank accession MN908947.3) [18]. Base-calling was performed in real-time by Guppy version 7.1.4 integrated within MinKNOW (Oxford Nanopore, UK) using high-accuracy base calling mode.

The RAMPART v1.2.0 software package developed by the ARTIC network was used to visualize mapping reads and genome coverage in real-time for each barcode [19]. Variant annotation, amplicon coverage analysis, Pango lineage, mutations and clade assignment were performed using Nextclade v3.2.0, EPI2ME and CZ ID [20–22]. Ambiguous bases (N) were introduced when the coverage depth was less than 10 reads, and these positions were excluded from the variant calling [23].

Maximum Likelihood phylogenetic trees were generated based on data stored on GISAID. The queried sequences were compared with the 16.8 million genomes in the EpiCoV database on GISAID to find related genomes [24]. These queried genomes passed quality control and met the minimum metadata requirements.

Before constructing the phylogenetic trees, we first retrieved genomes closely related to our sequences of interest from EpiCoV database using the Audacity Instant search algorithm [25]. The search was performed using standard parameters, ensuring that only relevant, high-quality matches were retained. The unique identifiers obtained were then mapped to the corresponding GISAID accessions and metadata. All selected genomes were compiled and prepared for subsequent phylogenetic analysis.

This analysis yielded 493 closely related genomes, including 67 from Côte d’Ivoire and 235 from African countries. All accessions IDs used are presented in supplementary Table 1.

Each phylogenetic tree was constructed in R (version 4.4.2) using the phangorn package. The sequences were first aligned with MAFFT, then the resulting alignment was imported in FASTA format. A distance matrix was calculated, and a starting tree was generated using the Neighbor-Joining (NJ) method. From this tree, a maximum likelihood model was constructed. The final tree was then exported in Newick format and imported into Interactive Tree of Life (iTOL version 7.2.2) for visualisation and annotation, including addition of different colour labels by lineage, city or country of origin [26].

Further analysis was executed in R [27]. Chi-square tests were used for categorical data to establish the relationship between SARS-CoV-2 carriage by geographical location, visits and clinical symptoms of infection. However, when at least one observed frequency was less than 5, we opted for Fisher’s exact test. Values of *p* < 0.05 were considered significant.

## Results

Out of 445 samples collected, 15 samples (3.37%) tested positive for SARS-CoV-2, 5 cases in Abidjan and 10 in Korhogo. The Ct values ranged from 23.67 to 34.13. For positive controls, Ct values ranged from 26.98 to 29.79. These samples were identified between February and April 2021 (from Survey 4 to 6). There were no significant differences in the prevalence of SARS-CoV-2 by geographical location (*p* = 0.27). There was a significant difference in SARS-CoV-2 carriage over time per sampling visit from February to April (*p* < 0.05). Pairwise comparisons of the prevalence of SARS-CoV-2 between all visits (S1-S6) are presented in Supplementary Table 2.

**Fig. 1:**
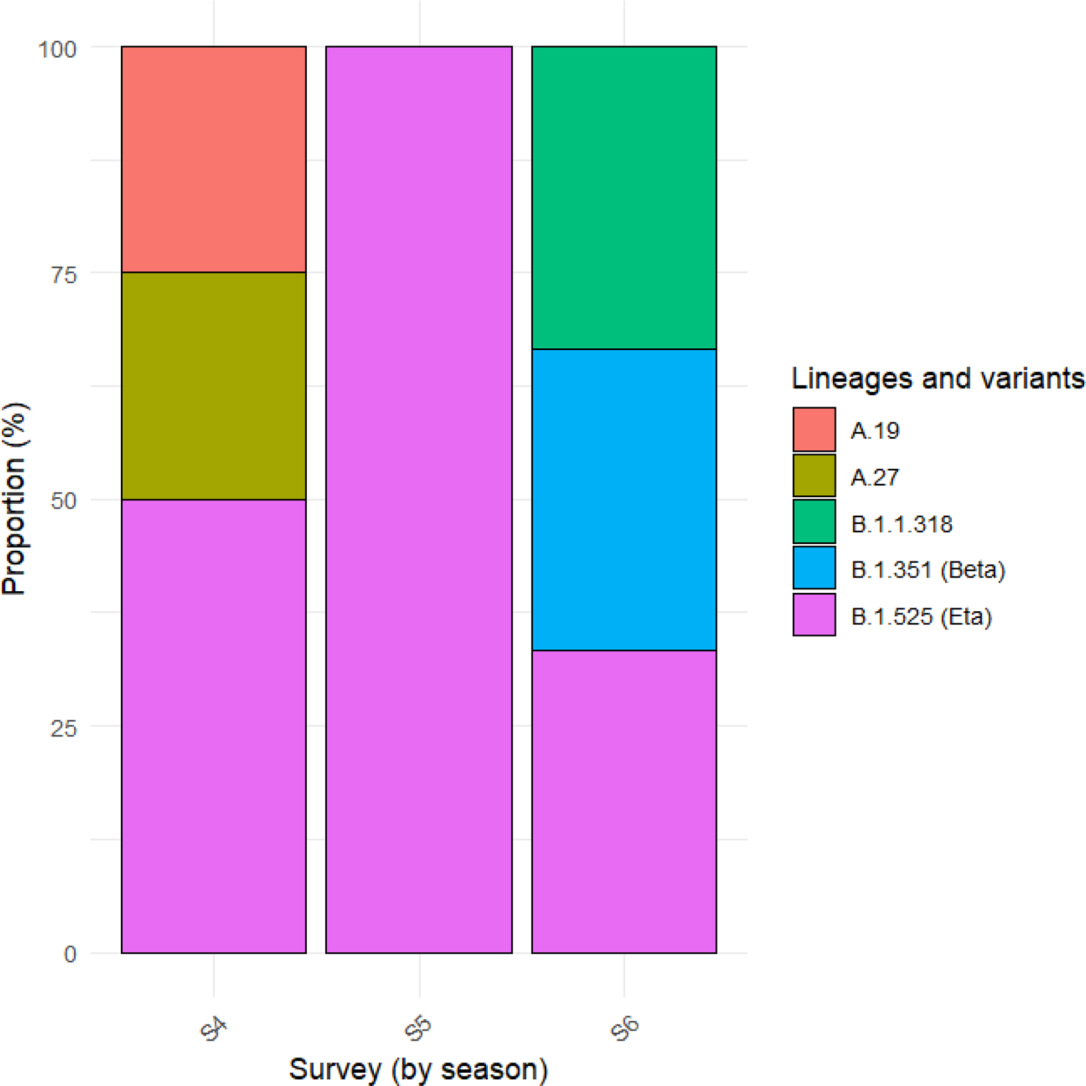
Proportion of SARS-CoV-2 lineages and variants identified by survey S4, S5, S6 represent the different months of virus detection (from February to April)

Genomic analyses showed that 12 out of 15 sequences (80%) had a genomic coverage of over 70%, and 10 out of 15 (66.7%) had coverage over 90%. Of these, 40% (6/15) were nearly complete genomes (missingness (N) < 500). Overall, lineage B.1.525 (*n* = 9, 60%), B.1.351 (*n* = 1, 6.7%), as well as other lineages such as B.1.1.318 (*n* = 1, 6.7%), A.19 (*n* = 2, 13.3%) and A.27 (*n* = 2, 13,3%) were the main variants identified. Supplementary Table 3 shows the whole list of variants identified. The different proportions of lineages and variants identified by survey are shown in Fig. 1.

The Eta variant was predominant at both study sites (60% in Korhogo, 60% in Abidjan) while the Beta variant was only identified in Abidjan. Among the other lineages, A.27 and A.19 were detected only in Korhogo (20%), while the B.1.1.318 type was detected only in Abidjan.

**Fig. 2:**
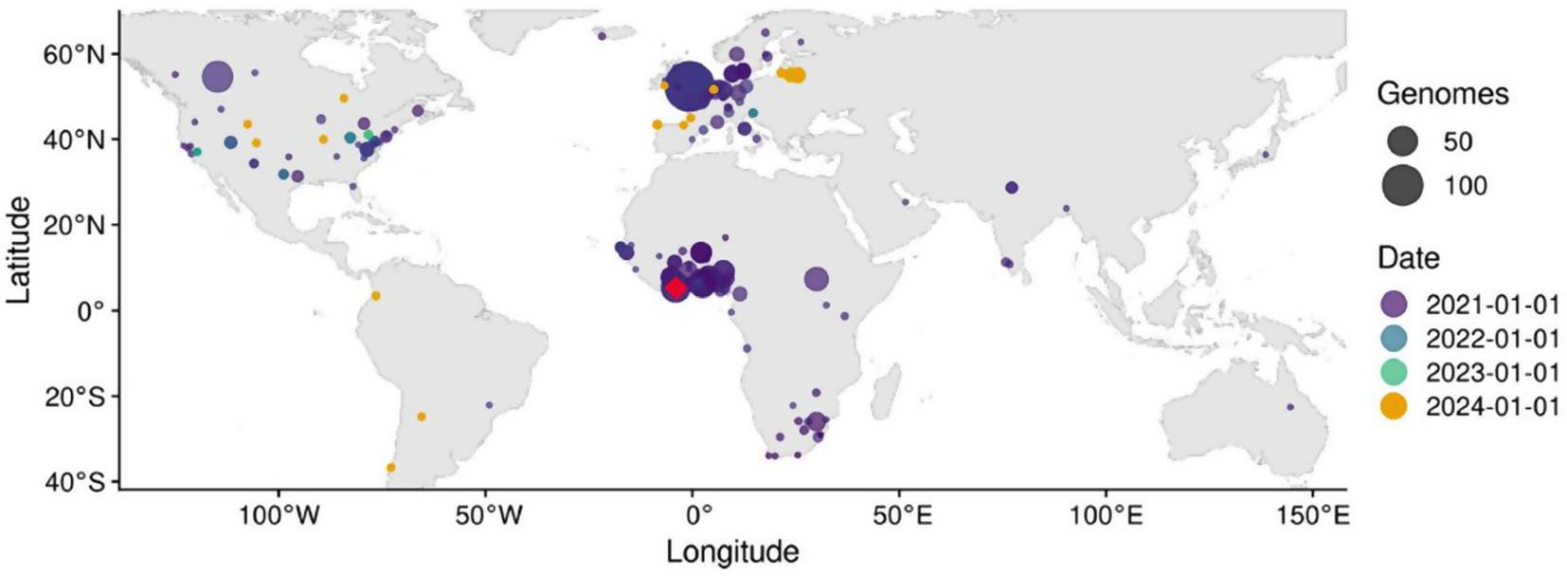
Locations of the related genomes and query sequences. The locations where related genomes were collected are shown on the map above. The size of the circle is related to the number of genomes; and the colour indicates the average collection date. All submitted sequences are indicated by a red diamond. The map was generated on GISAID

The analysis carried out on Global Initiative on Sharing All Influenza Data (GISAID) showed similarities with variants identified around the world (Fig. 2).

**Fig. 3:**
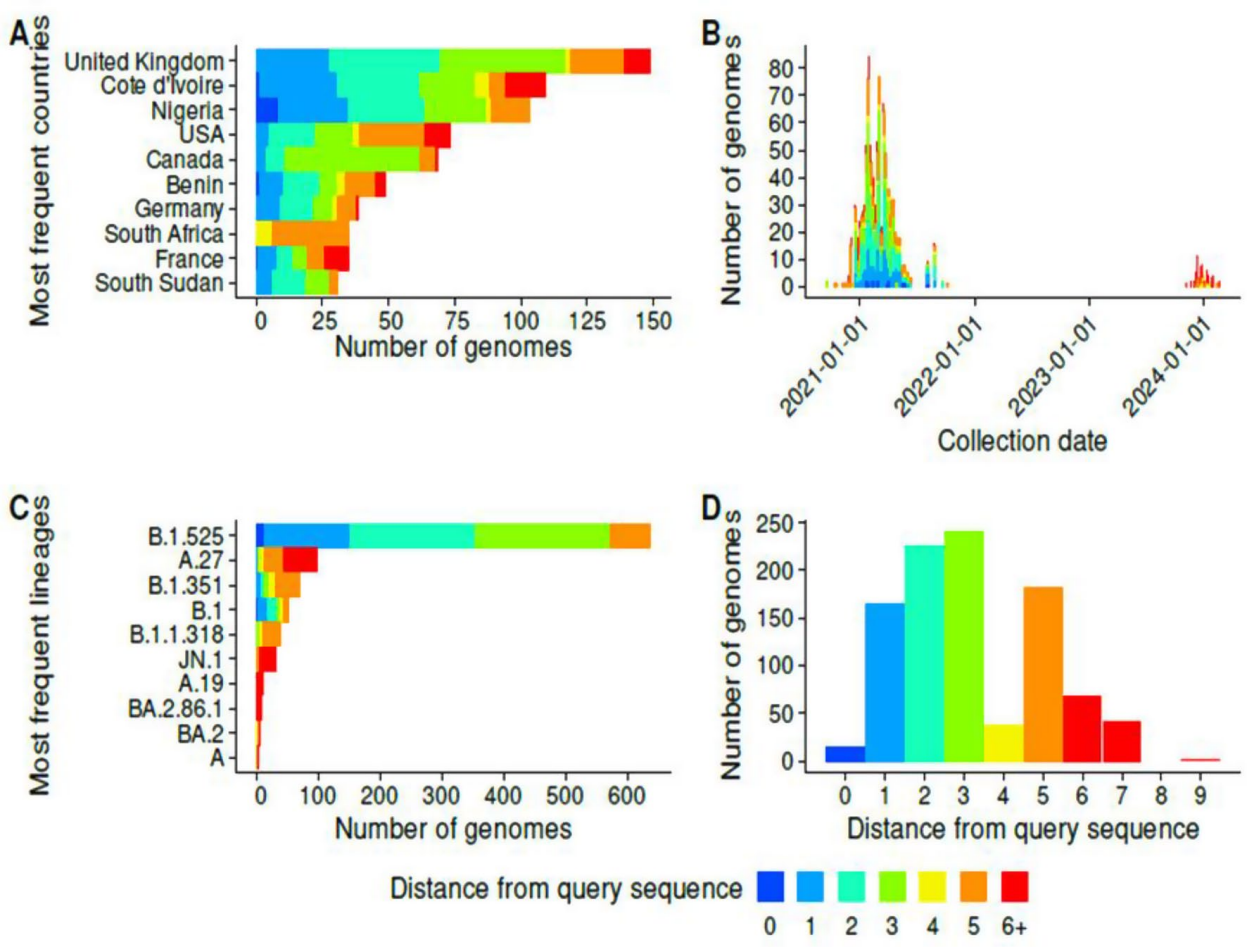
Characteristics of the related genomes. The related genomes are shown coloured by distance to the nearest query sequence. The charts show the countries with the highest number of genomes related to the study sequences (**A**), the number of related genomes by collection date (**B**), the number of related genomes assigned to each of the most frequent lineages (**C**), and the number of related genomes by distance from the closest query sequence (**D**)

Countries containing a large number of sequences resembling the query sequences were as follows: United Kingdom, Côte d’Ivoire, Nigeria, Canada, United States, Benin, Germany, South Africa, France and South Sudan (Fig. 3).

Five genomes related to lineage A.19 sequences were found after alignment, and they all came from Côte d’Ivoire. The list of genomes related to the other sequences is presented in Supplementary Table 4.

**Fig. 4:**
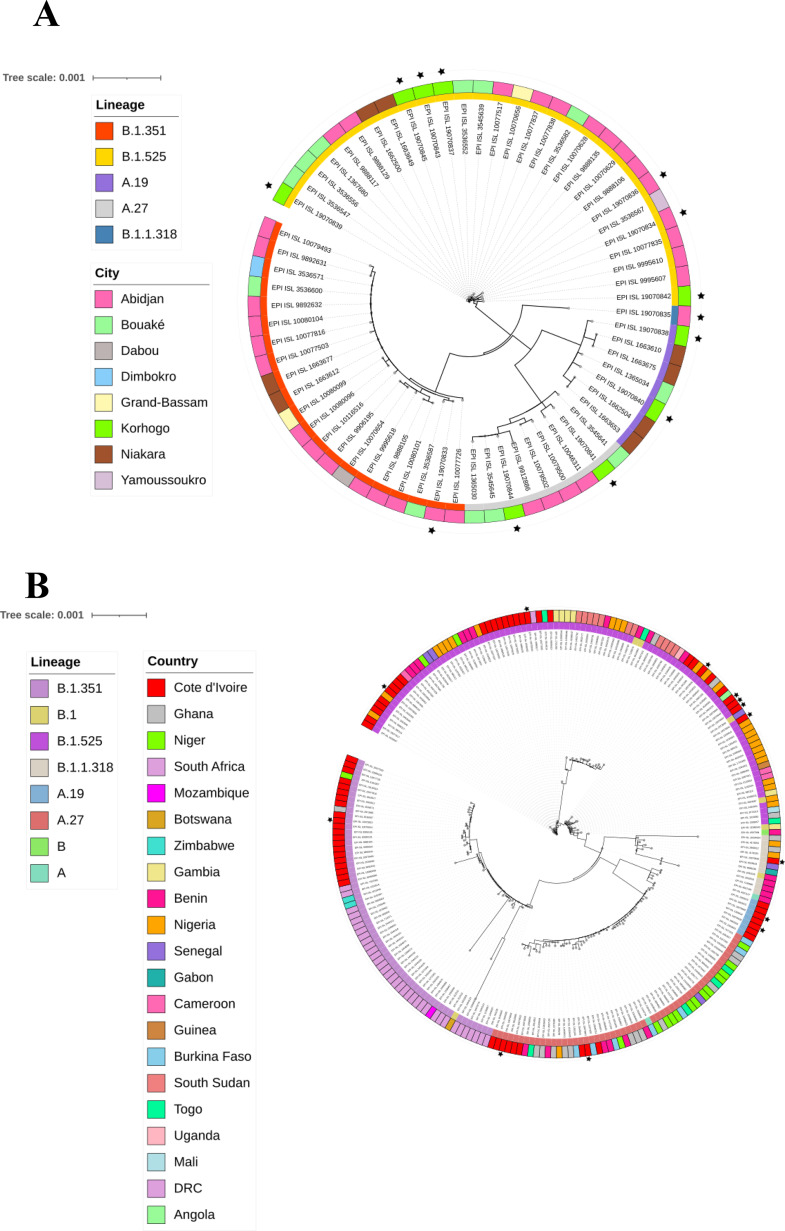
Maximum likelihood phylogenetic tree of the sequences analysed and the associated genomes from Côte d’Ivoire **(A)** and other African countries **(B).** The study sequences are represented in brown with black stars on each tree

All sequenced genomes were phylogenetically compared with representative genomes from Côte d’Ivoire, Africa (Fig. 4), as well as other countries around the world (supplementary Fig. 1).

Our study sequences show a polyphyletic distribution within the tree, overlapping with isolates from eight Ivorian cities and multiple African countries lineage B.1.525 is the most highly represented, with sequences mainly from Abidjan, Bouaké, and Niakara. Several of our sequences are associated with this lineage, forming clusters characterised by short branch lengths, indicating recent genetic proximity. Lineages B.1.351, A.27 and A.19 have a more restricted distribution, with some of our sequences placed close to isolates from Bouaké, Abidjan and Niakara. Comparative analysis reveals that our sequences do not form a single monophyletic cluster but are distributed across several lineages, suggesting multiple independent introductions of SARS-CoV-2 in Côte d’Ivoire.

Regarding the African phylogenetic tree, lineages B.1.525 and B.1.351 have the widest geographical distribution, with a substantial representation of sequences from South Africa, Côte d’Ivoire, Nigeria, Niger, and Benin. Our sequences appear frequently in these lineages, forming clusters mainly with isolates from Côte d’Ivoire, characterised by short branch lengths. Lineages B.1.1.318, A.27 and A.19 have a more moderate distribution, with some of our sequences positioned close to West African isolates, particularly those from Côte d’Ivoire, Burkina Faso and Nigeria.

**Fig. 5:**
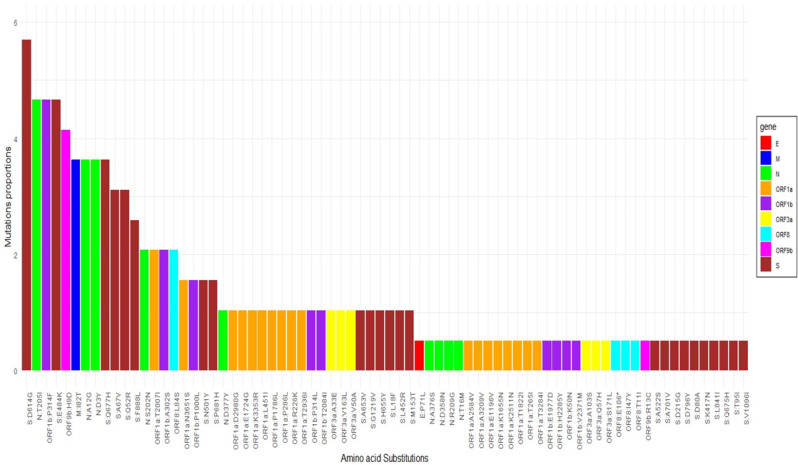
Representation of amino acid substitutions in viral proteins. Proportions of amino acid substitutions for all SARS-CoV-2 proteins taking into account all sequenced samples. Mutations were classified by gene (E, M, N, ORF1a, ORF1b, ORF3a, ORF8, ORF9b et S)

A total of 74 substitution types were characterised on viral proteins. Of these, the D614G mutation (*n* = 11/15, 73.33%) located on the S gene was the most common, followed by the T205I (*n* = 8/15, 53.33%) located on the N gene and P314F (*n* = 7/15, 46,66%) located on the ORF1b gene (Fig. 5).

## Discussion

Genomic surveillance of SARS-Cov-2 in children, particularly in developing countries, is very rare, as the various studies focused on adults [28]. This lack of interest exists, despite evidence that children can be infected and may experience milder symptoms, making them less likely to be identified through symptom-based viral testing [29]. This study is the first to report on the genomic characterisation of SARS-CoV-2 in children in Côte d’Ivoire using next-generation sequencing (ONT MinION).

The role of children in the transmission of COVID-19 has been debated worldwide [30]. A study by Jiehao et al. on children diagnosed with COVID-19 indicates that the potential risk of transmission from infected children to adult contacts should not be overlooked [31]. In our study, SARS-CoV-2 carriage varied significantly over time, with cases detected from S4 (February) to S6 (April) 2021 towards the end of the dry season. The period of detection of the positive samples in this study coincides with a period of high prevalence of the virus in Côte d’Ivoire according to the WHO [32]. These results reinforce the idea that transmission of the virus from children to family members should not be neglected in the context of an epidemic.

The different variants identified show changes that highlight genetic markers affecting disease severity, immune escape and therapeutic resistance [12, 13].

Of the 74 types of substitution obtained on viral proteins, the D614G mutation (73,33%) located in the S gene was the most common. This is similar to the results of a study conducted in Ghana [10]. This mutation has been shown to promote viral replication and infectivity, potentially increasing transmission. Plante et al.., have shown that it is also associated with serious cases of COVID-19 [33]. This highlights the need to monitor carriage in children in order to identify this VOI and better protect the population against COVID-19. This study was carried out before the emergence and widespread detection of the Delta variant in Côte d’Ivoire. Although the Delta variant began to be identified towards the end of the sample collection period, its presence was not yet relevant in the country.

The B.1.1.318 lineage had also been identified in a previous study conducted in Côte d’Ivoire, as well as in other African countries, including the Republic of Gabon and Ghana [34, 35]. This lineage, although not included in the VOC or VOI, has been of public health significance, as it was at the origin of the second wave in Mauritius, and was associated with high transmission and impaired vaccine efficacy [36].

Sequences belonging to lineage A.27 were also identified in a study conducted in five African countries, namely Niger, Tunisia, Senegal, Cameroon and Algeria [37].

Interestingly, related lineage A.19 genomes represented on the trees came only from central and northern Côte d’Ivoire (Bouaké and Niakara), with a total of 5 sequences. However, additional sequences from A.19 lineage were found in the GISAID database, including 113 from West African countries (Nigeria, Ghana, Côte d’Ivoire and Burkina Faso) as well as 16 from other regions of the world, deposited during the study period. This suggests that lineage A.19 circulated more frequently in the sub-region during the study period.

Hence the importance of closely monitoring the emergence of new variants, particularly in specific regions where they may spread undetected as they did in this case. This study identified carriage of that lineage dating from 2021. A greater number of carriage studies are needed to characterise the true diversity of the virus, including lineages currently underrepresented in cases but spreading among the population.

Although participants remained asymptomatic throughout the study, previous research has indicated that even asymptomatic carriers may experience long COVID, with development of autoimmune disorders or organ damages [38, 39]. This aspect was beyond the scope of the present study but warrants further investigation in future research.

Beyond viral diversity, studies on transmission dynamics have shown that the home environment was a key factor in virus spread and genomic diversity, highlighting the role of strict isolation and appropriate household management in limiting secondary transmission and promoting recovery [40].

Indeed, comparisons between pre-Delta and Delta strains revealed that Delta accumulated mutations more rapidly and followed a different evolutionary pathway, highlighting the need to interpret our results in the context of these overall evolutionary dynamics. Linking Delta’s rapid evolution to the study of pre-Delta variants provides a more complete view of how SARS-CoV-2 has adapted to selective pressures over time [41, 42].

Our study was able to detect a limited number of SARS-CoV-2 variants, with some variants being detected only once. This small sample size of positive samples limits our ability to draw definitive conclusions regarding the overall distribution of variants, their geographical specificity or temporal trends. The frequency of sampling (once a month at each site) may have limited our ability to detect SARS-CoV-2 carriers during certain periods leading to a limited number of positive.

Therefore, our results may not fully reflect the complete diversity and dynamics of SARS-CoV-2 circulation in the population studied.

Despite the small number of sequences generated, this retrospective study shows the importance of genomic surveillance of pathogens such as SARS-CoV-2 in carriage samples to better understand the epidemiology of the virus and prepare appropriate public health responses.

## Conclusion

This work characterizes SARS-CoV-2 carriage in a cohort of schoolchildren followed from November 2020 to April 2021 in Côte d’Ivoire using Oxford Nanopore Technology. The results highlight the diversity of SARS-CoV-2 variants, which in comparison were found in several countries around the world. In this study, SARS-CoV-2 carriage varied significantly over time with cases detected from S4 (February) to S6 (April) towards the end of the dry season. The identified sequences were from lineage B.1.525, A.19, A.27, B.1.1.318 and B.1.351. The SARS-CoV-2 lineage A.19, detected in two carriers in this study, also showed notable spread in the subregion compared to other sequences deposited during the study period.

This underlines the importance of pathogen surveillance in carriers and research into the evolution of the virus to guide public health strategies.

## Supplementary Information

Below is the link to the electronic supplementary material.

Supplementary Material 1

Supplementary Material 2

Supplementary Material 3

Supplementary Material 4

Supplementary Material 5

Supplementary Material 6

## Data Availability

The raw sequences have been shared on Global Initiative on Sharing All Influenza Data (GISAID) database and can be accessed by registered GISAID users under the accession numbers EPI_ISL_19070833, EPI_ISL_19070834, EPI_ISL_19070835, EPI_ISL_19070836, EPI_ISL_19070837, EPI_ISL_19070838, EPI_ISL_19070839, EPI_ISL_19070840, EPI_ISL_19070841, EPI_ISL_19070842, EPI_ISL_19070843, EPI_ISL_19070844, and EPI_ISL_19070845.The following doi: [10.55876/gis8.240425uq](10.55876/gis8.240425uq) and Supplementary material 1 contain all information such as accession number, virus name, collection date and submitting lab. All the accession IDs for the other sequences used are available on GISAID and are shown in supplementary Table 1.

## Abbreviations

COVID-19: Coronavirus disease 2019
CSRS: Centre Suisse de Recherches Scientifiques en Côte d’Ivoire
GISAID: Global Initiative on Sharing All Influenza Data
SARS-CoV-2: Severe acute respiratory syndrome coronavirus 2
UAR: Unité d’Appui à la Recherche
VOC: Variants of concern VOC
VOI: Variants of interest VOI
WACCBIP: West African Centre for Cell Biology of Infectious Pathogens
WGS: Whole genome sequencing
WHO: World Health Organization
