# Supplementary material for: Genomic diversity of SARS-CoV-2 carriage in a cohort of schoolchildren in Côte d’ivoire during COVID-19 pandemics: insights from pre-delta emergence
Source: BMC Infect Dis. 2026 Jan 8;26:261. doi: 10.1186/s12879-025-12374-4 (PMC12870052; doi:10.1186/s12879-025-12374-4)
Supplement: Supplementary file 2 — Supplementary Material 2 [file 12879_2025_12374_MOESM2_ESM.docx]

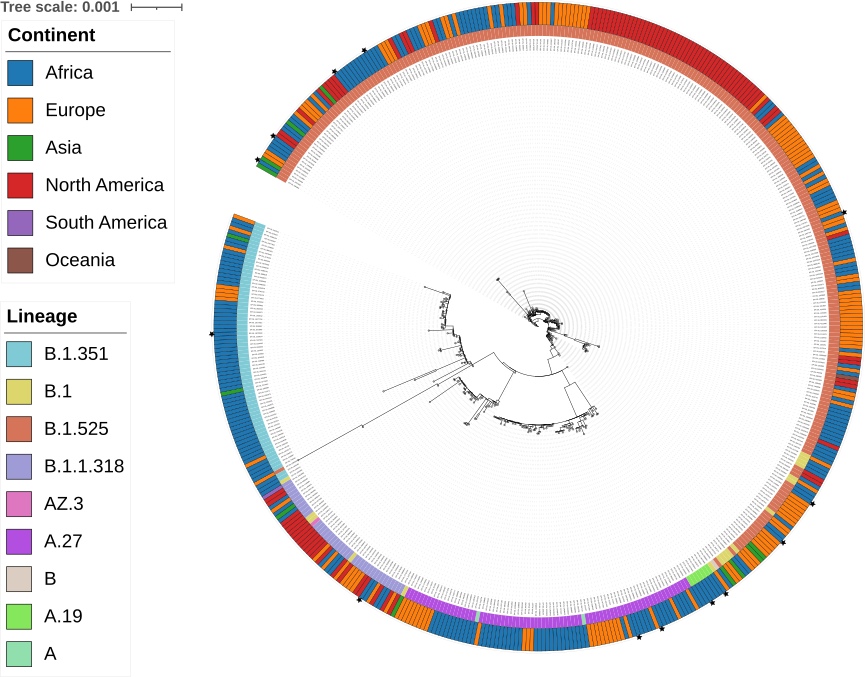


**S1 figure.** Maximum likelihood phylogenetic tree of the query sequences and related genomes worldwide.

Our study sequences are distributed in several distinct phylogenetic groups, demonstrating significant genetic diversity within our sample. Comparative analysis with international reference sequences reveals preferential phylogenetic associations with isolates from the African continent, as shown by the strong predominance of African sequences (in blue) in the tree, followed by notable representation from Europe (in orange) and North America (in red).
