# Supplementary material for: Genomic diversity of SARS-CoV-2 carriage in a cohort of schoolchildren in Côte d’ivoire during COVID-19 pandemics: insights from pre-delta emergence
Source: BMC Infect Dis. 2026 Jan 8;26:261. doi: 10.1186/s12879-025-12374-4 (PMC12870052; doi:10.1186/s12879-025-12374-4)
Supplement: Supplementary file 3 — Supplementary Material 3 [file 12879_2025_12374_MOESM3_ESM.docx]

**S1 table:** GISAID accession IDs of closely related SARS-CoV-2 genomes included in the maximum likelihood trees.

| **Accession ID** | **Accession ID** | **Accession ID** | **Accession ID** | **Accession ID** | **Accession ID** | **Accession ID** | **Accession ID** | **Accession ID** | **Accession ID** | **Accession ID** | **Accession ID** | **Accession ID** | **Accession ID** | **Accession ID** |
| --- | --- | --- | --- | --- | --- | --- | --- | --- | --- | --- | --- | --- | --- | --- |
| EPI_ISL_1272301 | EPI_ISL_2658698 | EPI_ISL_2169508 | EPI_ISL_2934315 | EPI_ISL_6492769 | EPI_ISL_1895626 | EPI_ISL_4471403 | EPI_ISL_916301 | EPI_ISL_1788788 | EPI_ISL_2376384 | EPI_ISL_4567108 | EPI_ISL_762449 | EPI_ISL_2264845 | EPI_ISL_9888129 | EPI_ISL_19070833 |
| EPI_ISL_3342396 | EPI_ISL_2169260 | EPI_ISL_2169244 | EPI_ISL_2968012 | EPI_ISL_4372598 | EPI_ISL_2164530 | EPI_ISL_4471377 | EPI_ISL_945750 | EPI_ISL_3800991 | EPI_ISL_10438423 | EPI_ISL_4567092 | EPI_ISL_1165571 | EPI_ISL_1610588 | EPI_ISL_3386207 | EPI_ISL_19070834 |
| EPI_ISL_7337581 | EPI_ISL_2169240 | EPI_ISL_2169311 | EPI_ISL_1667047 | EPI_ISL_1991991 | EPI_ISL_2934213 | EPI_ISL_1300334 | EPI_ISL_16520578 | EPI_ISL_15570857 | EPI_ISL_801441 | EPI_ISL_989095 | EPI_ISL_10070656 | EPI_ISL_2650845 | EPI_ISL_9995607 | EPI_ISL_19070835 |
| EPI_ISL_1191948 | EPI_ISL_2169379 | EPI_ISL_2170448 | EPI_ISL_2204804 | EPI_ISL_1991990 | EPI_ISL_2467678 | EPI_ISL_2958671 | EPI_ISL_2450798 | EPI_ISL_3545645 | EPI_ISL_801442 | EPI_ISL_1197062 | EPI_ISL_8997325 | EPI_ISL_1367680 | EPI_ISL_998080 | EPI_ISL_19070836 |
| EPI_ISL_2179366 | EPI_ISL_2169549 | EPI_ISL_2169545 | EPI_ISL_4178281 | EPI_ISL_7139680 | EPI_ISL_1884134 | EPI_ISL_6308148 | EPI_ISL_16520580 | EPI_ISL_1333887 | EPI_ISL_1443910 | EPI_ISL_8065534 | EPI_ISL_10077837 | EPI_ISL_2450805 | EPI_ISL_10438535 | EPI_ISL_19070837 |
| EPI_ISL_1191907 | EPI_ISL_2170205 | EPI_ISL_2169880 | EPI_ISL_1376828 | EPI_ISL_4965219 | EPI_ISL_1872095 | EPI_ISL_2504496 | EPI_ISL_2346435 | EPI_ISL_1365030 | EPI_ISL_3804062 | EPI_ISL_2693667 | EPI_ISL_1145722 | EPI_ISL_2861328 | EPI_ISL_10438432 | EPI_ISL_19070841 |
| EPI_ISL_2421131 | EPI_ISL_2169917 | EPI_ISL_2477817 | EPI_ISL_1440915 | EPI_ISL_4377655 | EPI_ISL_1366579 | EPI_ISL_4567099 | EPI_ISL_1457613 | EPI_ISL_4567105 | EPI_ISL_1508990 | EPI_ISL_1263898 | EPI_ISL_1145742 | EPI_ISL_2450811 | EPI_ISL_2227203 | EPI_ISL_19070842 |
| EPI_ISL_9888105 | EPI_ISL_2169236 | EPI_ISL_2163824 | EPI_ISL_2389920 | EPI_ISL_2389919 | EPI_ISL_919859 | EPI_ISL_2450801 | EPI_ISL_1301960 | EPI_ISL_985238 | EPI_ISL_10079500 | EPI_ISL_2720404 | EPI_ISL_978226 | EPI_ISL_1434170 | EPI_ISL_1591100 | EPI_ISL_19070844 |
| EPI_ISL_660659 | EPI_ISL_2169557 | EPI_ISL_2170458 | EPI_ISL_1585940 | EPI_ISL_12698065 | EPI_ISL_1668836 | EPI_ISL_944748 | EPI_ISL_941289 | EPI_ISL_10438444 | EPI_ISL_1364949 | EPI_ISL_2450803 | EPI_ISL_966312 | EPI_ISL_1439712 | EPI_ISL_10438449 | EPI_ISL_19070838 |
| EPI_ISL_2153514 | EPI_ISL_2169367 | EPI_ISL_1731555 | EPI_ISL_10334614 | EPI_ISL_17160260 | EPI_ISL_2433104 | EPI_ISL_1251931 | EPI_ISL_2672210 | EPI_ISL_811127 | EPI_ISL_2164509 | EPI_ISL_1226710 | EPI_ISL_10077517 | EPI_ISL_855634 | EPI_ISL_7477220 | EPI_ISL_19070839 |
| EPI_ISL_10080101 | EPI_ISL_2169336 | EPI_ISL_11586548 | EPI_ISL_2769242 | EPI_ISL_3908610 | EPI_ISL_919872 | EPI_ISL_1251906 | EPI_ISL_1457575 | EPI_ISL_9912886 | EPI_ISL_1862587 | EPI_ISL_2450781 | EPI_ISL_1035823 | EPI_ISL_9888117 | EPI_ISL_10438554 | EPI_ISL_19070840 |
| EPI_ISL_2467708 | EPI_ISL_2169552 | EPI_ISL_2170335 | EPI_ISL_8506411 | EPI_ISL_1541129 | EPI_ISL_1605311 | EPI_ISL_4113473 | EPI_ISL_1457574 | EPI_ISL_1508959 | EPI_ISL_8073937 | EPI_ISL_2308268 | EPI_ISL_1077044 | EPI_ISL_1365034 | EPI_ISL_1749293 | EPI_ISL_19070843 |
| EPI_ISL_2467701 | EPI_ISL_2169252 | EPI_ISL_1300849 | EPI_ISL_4360171 | EPI_ISL_2167327 | EPI_ISL_1643712 | EPI_ISL_1251897 | EPI_ISL_1457576 | EPI_ISL_10079502 | EPI_ISL_961609 | EPI_ISL_1887271 | EPI_ISL_1035819 | EPI_ISL_1663653 | EPI_ISL_2281778 | EPI_ISL_19070845 |
| EPI_ISL_2467699 | EPI_ISL_2169358 | EPI_ISL_11586547 | EPI_ISL_2107644 | EPI_ISL_2167309 | EPI_ISL_8528317 | EPI_ISL_4567094 | EPI_ISL_1737890 | EPI_ISL_17746322 | EPI_ISL_1493017 | EPI_ISL_14205942 | EPI_ISL_2932535 | EPI_ISL_1662504 | EPI_ISL_2958658 |  |
| EPI_ISL_1787370 | EPI_ISL_2169496 | EPI_ISL_12388389 | EPI_ISL_8528533 | EPI_ISL_1870095 | EPI_ISL_2164507 | EPI_ISL_4112665 | EPI_ISL_4026324 | EPI_ISL_10438402 | EPI_ISL_3914919 | EPI_ISL_2450777 | EPI_ISL_7139676 | EPI_ISL_1663675 | EPI_ISL_1749312 |  |
| EPI_ISL_1130868 | EPI_ISL_2169877 | EPI_ISL_7139586 | EPI_ISL_4964183 | EPI_ISL_2167305 | EPI_ISL_8528330 | EPI_ISL_3914993 | EPI_ISL_1675031 | EPI_ISL_3914952 | EPI_ISL_2167331 | EPI_ISL_1410240 | EPI_ISL_4567020 | EPI_ISL_1663610 | EPI_ISL_1749335 |  |
| EPI_ISL_11586544 | EPI_ISL_2479114 | EPI_ISL_982299 | EPI_ISL_4382035 | EPI_ISL_11586531 | EPI_ISL_2551373 | EPI_ISL_1380528 | EPI_ISL_1821601 | EPI_ISL_2958659 | EPI_ISL_1716810 | EPI_ISL_1831442 | EPI_ISL_1970553 | EPI_ISL_4962540 | EPI_ISL_3545641 |  |
| EPI_ISL_2467693 | EPI_ISL_2169380 | EPI_ISL_1091671 | EPI_ISL_4374740 | EPI_ISL_1706505 | EPI_ISL_1566076 | EPI_ISL_2450804 | EPI_ISL_1232273 | EPI_ISL_10438433 | EPI_ISL_3086917 | EPI_ISL_1333101 | EPI_ISL_2432954 | EPI_ISL_1706496 | EPI_ISL_2227199 |  |
| EPI_ISL_10077835 | EPI_ISL_2170201 | EPI_ISL_7139594 | EPI_ISL_4375013 | EPI_ISL_10010859 | EPI_ISL_7477201 | EPI_ISL_2450799 | EPI_ISL_1508957 | EPI_ISL_2281827 | EPI_ISL_1242024 | EPI_ISL_1376799 | EPI_ISL_1381832 | EPI_ISL_3536556 | EPI_ISL_10438409 | |
| EPI_ISL_9888135 | EPI_ISL_2169553 | EPI_ISL_9995610 | EPI_ISL_14813892 | EPI_ISL_2460861 | EPI_ISL_2227200 | EPI_ISL_2450782 | EPI_ISL_1821602 | EPI_ISL_12240055 | EPI_ISL_1263993 | EPI_ISL_760883 | EPI_ISL_2432050 | EPI_ISL_7219482 | EPI_ISL_827463 |  |
| EPI_ISL_10070629 | EPI_ISL_2169368 | EPI_ISL_3536582 | EPI_ISL_17746710 | EPI_ISL_2734861 | EPI_ISL_2958663 | EPI_ISL_3536567 | EPI_ISL_1165569 | EPI_ISL_2281813 | EPI_ISL_1235655 | EPI_ISL_797195 | EPI_ISL_2117448 | EPI_ISL_3536552 | EPI_ISL_4567137 |  |
| EPI_ISL_10070628 | EPI_ISL_2478434 | EPI_ISL_2740740 | EPI_ISL_8886330 | EPI_ISL_3545639 | EPI_ISL_757470 | EPI_ISL_1118444 | EPI_ISL_985110 | EPI_ISL_2281839 | EPI_ISL_1263938 | EPI_ISL_985121 | EPI_ISL_7220038 | EPI_ISL_1662500 | EPI_ISL_13531871 | |
| EPI_ISL_2166335 | EPI_ISL_2478431 | EPI_ISL_1473516 | EPI_ISL_4374271 | EPI_ISL_1545914 | EPI_ISL_757974 | EPI_ISL_1192251 | EPI_ISL_991220 | EPI_ISL_2281798 | EPI_ISL_1871512 | EPI_ISL_4026407 | EPI_ISL_2967031 | EPI_ISL_1663649 | EPI_ISL_2142724 |  |
| EPI_ISL_2167323 | EPI_ISL_2163823 | EPI_ISL_1504832 | EPI_ISL_4963816 | EPI_ISL_928731 | EPI_ISL_10046311 | EPI_ISL_1314407 | EPI_ISL_8997332 | EPI_ISL_2281799 | EPI_ISL_3120638 | EPI_ISL_1165570 | EPI_ISL_3133652 | EPI_ISL_4567120 | EPI_ISL_1575442 |  |
| EPI_ISL_2165796 | EPI_ISL_2169324 | EPI_ISL_10077838 | EPI_ISL_4963076 | EPI_ISL_1212980 | EPI_ISL_10438541 | EPI_ISL_12828399 | EPI_ISL_4112616 | EPI_ISL_2281777 | EPI_ISL_11586543 | EPI_ISL_1263933 | EPI_ISL_1715399 | EPI_ISL_2450790 | EPI_ISL_3804035 |  |
| EPI_ISL_2165788 | EPI_ISL_2169262 | EPI_ISL_2505980 | EPI_ISL_4379134 | EPI_ISL_1707340 | EPI_ISL_10438435 | EPI_ISL_1000632 | EPI_ISL_9888106 | EPI_ISL_1749373 | EPI_ISL_2281795 | EPI_ISL_1263953 | EPI_ISL_2873836 | EPI_ISL_985114 | EPI_ISL_3804060 |  |
| EPI_ISL_2167328 | EPI_ISL_2169319 | EPI_ISL_1377087 | EPI_ISL_3115790 | EPI_ISL_2164508 | EPI_ISL_4471392 | EPI_ISL_999181 | EPI_ISL_1371287 | EPI_ISL_2958667 | EPI_ISL_1178019 | EPI_ISL_1409202 | EPI_ISL_3536547 | EPI_ISL_985111 | EPI_ISL_3804031 |  |
| EPI_ISL_2166361 | EPI_ISL_2169320 | EPI_ISL_4178333 | EPI_ISL_4567169 | EPI_ISL_3410943 | EPI_ISL_4471406 | EPI_ISL_1333968 | EPI_ISL_2188489 | EPI_ISL_1749368 | EPI_ISL_1332876 | EPI_ISL_1411637 | EPI_ISL_1832617 | EPI_ISL_985112 | EPI_ISL_3804019 |  |
| EPI_ISL_2165727 | EPI_ISL_2170211 | EPI_ISL_2460582 | EPI_ISL_2425080 | EPI_ISL_16139954 | EPI_ISL_4471413 | EPI_ISL_937978 | EPI_ISL_1018082 | EPI_ISL_1749372 | EPI_ISL_1910867 | EPI_ISL_1451027 | EPI_ISL_1832588 | EPI_ISL_942353 | EPI_ISL_1093451 |  |
| EPI_ISL_933199 | EPI_ISL_2163820 | EPI_ISL_1440924 | EPI_ISL_2932553 | EPI_ISL_3988365 | EPI_ISL_2359858 | EPI_ISL_916226 | EPI_ISL_7139635 | EPI_ISL_3804061 | EPI_ISL_17547559 | EPI_ISL_2006571 | EPI_ISL_1235665 | EPI_ISL_2677789 | EPI_ISL_2893235 |  |
