# Supplementary material for: Genomic diversity of SARS-CoV-2 carriage in a cohort of schoolchildren in Côte d’ivoire during COVID-19 pandemics: insights from pre-delta emergence
Source: BMC Infect Dis. 2026 Jan 8;26:261. doi: 10.1186/s12879-025-12374-4 (PMC12870052; doi:10.1186/s12879-025-12374-4)
Supplement: Supplementary file 4 — Supplementary Material 4 [file 12879_2025_12374_MOESM4_ESM.docx]

**S2 table**: Pairwise comparisons of the prevalence of SARS-CoV-2 between all visits (S1-S6)

| **Comparaison** | **Test** | **OR** | **95% CI** | **p_value** |
| --- | --- | --- | --- | --- |
| S1 vs S2 | Chi-squared | NA | NA | 1 |
| S1 vs S3 | Chi-squared | NA | NA | 1 |
| S1 vs S4 | Fisher exact | Inf | 1.81-Inf | 0.00642 |
| S1 vs S5 | Fisher exact | Inf | 0.69-Inf | 0.05672 |
| S1 vs S6 | Fisher exact | Inf | 0.43-Inf | 0.11499 |
| S2 vs S3 | Chi-squared | NA | NA | 1 |
| S2 vs S4 | Fisher exact | Inf | 1.82-Inf | 0.00642 |
| S2 vs S5 | Fisher exact | Inf | 0.69-Inf | 0.05672 |
| S2 vs S6 | Fisher exact | Inf | 0.43-Inf | 0.11499 |
| S3 vs S4 | Fisher exact | Inf | 1.82-Inf | 0.00649 |
| S3 vs S5 | Fisher exact | Inf | 0.69-Inf | 0.05677 |
| S3 vs S6 | Fisher exact | Inf | 0.43-Inf | 0.11499 |
| S4 vs S5 | Fisher exact | 0.49 | 0.10-1.92 | 0.36777 |
| S4 vs S6 | Fisher exact | 0.37 | 0.06-1.6 | 0.20967 |
| S5 vs S6 | Fisher exact | 0.75 | 0.10-4.62 | 1 |
