# Supplementary material for: Genomic diversity of SARS-CoV-2 carriage in a cohort of schoolchildren in Côte d’ivoire during COVID-19 pandemics: insights from pre-delta emergence
Source: BMC Infect Dis. 2026 Jan 8;26:261. doi: 10.1186/s12879-025-12374-4 (PMC12870052; doi:10.1186/s12879-025-12374-4)
Supplement: Supplementary file 5 — Supplementary Material 5 [file 12879_2025_12374_MOESM5_ESM.docx]

**S3 table**: Lineages and variants identified

| **ID** | **Ct value** | **N-count** | **Coverage (%)** | **Pango lineage** | **variants** | **Total reads** | **Mapped reads** | **Total nt Substitutions** | **Total nt Deletions** | **Total nt Insertions** | **Total FrameShifts** | **Total AA Substitutions** | **Total AA Deletions** |
| --- | --- | --- | --- | --- | --- | --- | --- | --- | --- | --- | --- | --- | --- |
| 03-0039-S4 | 23.67 | 126 | 99.6 | B.1.525 | Eta | 49399 | 43866 | 29 | 74 | 0 | 1 | 18 | 9 |
| 03-0016-S5 | 26.38 | 126 | 99.6 | B.1.525 | Eta | 41873 | 37550 | 27 | 74 | 0 | 1 | 14 | 9 |
| 03-0008-S6 | 28.34 | 347 | 98,86 | B.1.351 | Beta | 60918 | 54635 | 25 | 18 | 0 | 0 | 17 | 6 |
| 03-0018-S6 | 24.83 | 1601 | 94.67 | B.1.1.318 |  | 38547 | 34704 | 25 | 21 | 0 | 0 | 16 | 5 |
| 03-0036-S6 | 24.48 | 25052 | 16.22 | B.1.525 | Eta | 1772 | 1223 | 2 | 0 | 0 | 0 | 2 | 0 |
| 04-0001-S4 | 34.13 | 9590 | 67.96 | B.1.525 | Eta | 5313 | 4321 | 18 | 70 | 0 | 0 | 10 | 7 |
| 04-0006-S4 | 24.75 | 1242 | 95.87 | A.19 |  | 12514 | 11352 | 26 | 0 | 0 | 0 | 13 | 0 |
| 04-0011-S4 | 25.61 | 126 | 99.6 | A.19 |  | 22001 | 20269 | 29 | 0 | 0 | 0 | 13 | 0 |
| 04-0018-S4 | 28.81 | 1077 | 96.42 | A.27 |  | 27906 | 25228 | 28 | 15 | 0 | 1 | 14 | 6 |
| 04-0029-S4 | 31.18 | 1625 | 94.59 | B.1.525 | Eta | 29486 | 25301 | 25 | 74 | 0 | 1 | 13 | 9 |
| 04-0034-S4 | 23.72 | 126 | 99.6 | A.27 |  | 42193 | 38164 | 30 | 15 | 0 | 1 | 14 | 6 |
| 04-0036-S4 | 24.46 | 126 | 99.6 | B.1.525 | Eta | 28394 | 25747 | 25 | 74 | 0 | 1 | 13 | 9 |
| 04-0010-S5 | 27.54 | 1613 | 94.63 | B.1.525 | Eta | 19228 | 17522 | 24 | 74 | 0 | 1 | 13 | 9 |
| 04-0028-S5 | 31.17 | 7382 | 75.34 | B.1.525 | Eta | 6892 | 6080 | 20 | 58 | 0 | 0 | 10 | 3 |
| 04-0038-S5 | 29.27 | 29551 | 1.18 | B.1.525 | Eta | 986 | 362 | 0 | 0 | 0 | 0 | 0 | 0 |

Nt: nucleotide

AA: amino acid
