# Supplementary material for: Genomic diversity of SARS-CoV-2 carriage in a cohort of schoolchildren in Côte d’ivoire during COVID-19 pandemics: insights from pre-delta emergence
Source: BMC Infect Dis. 2026 Jan 8;26:261. doi: 10.1186/s12879-025-12374-4 (PMC12870052; doi:10.1186/s12879-025-12374-4)
Supplement: Supplementary file 6 — Supplementary Material 6 [file 12879_2025_12374_MOESM6_ESM.docx]

**S4 table**: List of study sequences and related genomes

| **Sequences** | **ID** | **GISAID ID** | **Related genomes** |
| --- | --- | --- | --- |
| Sequence 1 hCoV-19 | 03-0008-S6 | EPI_ISL_19070833 | 71 |
| Sequence 2 hCoV-19 | 03-0016-S5 | EPI_ISL_19070834 | 138 |
| Sequence 3 hCoV-19 | 03-0018-S6 | EPI_ISL_19070835 | 47 |
| Sequence 4 hCoV-19 | 03-0039-S4 | EPI_ISL_19070836 | 71 |
| Sequence 5 hCoV-19 | 04-0001-S4 | EPI_ISL_19070837 | 51 |
| Sequence 6 hCoV-19 | 04-0006-S4 | EPI_ISL_19070838 | 5 |
| Sequence 7 hCoV-19 | 04-0010-S5 | EPI_ISL_19070839 | 118 |
| Sequence 8 hCoV-19 | 04-0011-S4 | EPI_ISL_19070840 | 5 |
| Sequence 9 hCoV-19 | 04-0018-S4 | EPI_ISL_19070841 | 42 |
| Sequence 10 hCoV-19 | 04-0028-S5 | EPI_ISL_19070842 | 124 |
| Sequence 11 hCoV-19 | 04-0029-S4 | EPI_ISL_19070843 | 95 |
| Sequence 12 hCoV-19 | 04-0034-S4 | EPI_ISL_19070844 | 61 |
| Sequence 13 hCoV-19 | 04-0036-S4 | EPI_ISL_19070845 | 86 |
